# Clinical Efficacy and Safety of Multiple High-Dose Antibiotic-Loaded Cement Spacers in the Two-Stage Revision of Gram-Positive Periprosthetic Joint Infection

**DOI:** 10.3390/microorganisms14040768

**Published:** 2026-03-27

**Authors:** Miguel Márquez-Gómez, Lourdes Prats-Peinado, José Antonio Matas Díaz, Mar Sánchez-Somolinos, María Guembe, Javier Vaquero, Pablo Sanz-Ruiz

**Affiliations:** 1 Department of Orthopaedic Surgery and Traumatology, Hospital General Universitario Gregorio Marañón, 28007 Madrid, Spain; marquez.gomez.miguel@gmail.com (M.M.-G.); lurpratspeinado@gmail.com (L.P.-P.); joseantonio.matas@salud.madrid.org (J.A.M.D.); vaquerocot@gmail.com (J.V.); 2 Department of Clinical Microbiology and Infectious Diseases, Hospital General Universitario Gregorio Marañón, 28007 Madrid, Spain; mssomolinos@salud.madrid.org (M.S.-S.); maria.guembe@iisgm.com (M.G.); 3 Instituto de Investigación Sanitaria Gregorio Marañón, 28007 Madrid, Spain; 4 Medicine Department, School of Medicine, Universidad Complutense de Madrid, 28040 Madrid, Spain

**Keywords:** periprosthetic joint infection, bone cement, antibiotic-loaded cement spacer, two-stage revision, Gram-positive, vancomycin, ceftazidime

## Abstract

Periprosthetic joint infection (PJI) is a devastating complication after total joint arthroplasty, typically managed through a two-stage revision protocol involving antibiotic-loaded spacers. This study aimed to evaluate the clinical outcomes and safety of a new multiple high-dose antibiotic-loaded cement (MHDALC) spacer against alternative classical antibiotic combinations for Gram-positive PJI. In this retrospective observational study of 102 patients (30 MHDALC vs. 72 control), the MHDALC cohort received spacers prepared with commercial cement (1 g clindamycin and 1 g gentamicin per 40 g) supplemented with manual additions of 4 g vancomycin and 2 g ceftazidime per 40 g of cement, while the control group received the same commercial cement supplemented with 4 g of vancomycin alone. Treatment failure was significantly lower in the MHDALC group (6.6%) compared to the control group (20.8%; *p* = 0.005). Furthermore, the time to second-stage reimplantation was drastically reduced in the MHDALC cohort (9.1 vs. 17.8 weeks; *p* = 0.001). Despite the substantially higher antibiotic load, no significant differences were observed regarding mechanical or surgical complications between the two groups (*p* = 1.00). In conclusion, the use of MHDALC spacers is an effective and safe strategy for treating Gram-positive PJI, significantly improving eradication rates and accelerating the transition to definitive reconstruction without compromising structural integrity.

## 1. Introduction

Periprosthetic joint infection (PJI) remains one of the most devastating complications following joint arthroplasty, significantly impacting patient quality of life and imposing a substantial burden on healthcare systems [1]. Currently, the two-stage revision protocol is widely considered the gold standard for managing chronic PJI. A critical component of this strategy is the use of an antibiotic-loaded cement spacer, which serves the dual purpose of maintaining joint space and delivering high local concentrations of antimicrobial agents to eradicate remaining pathogens.

The combination of vancomycin and gentamicin in a cement spacer is commonly used [2,3,4,5]. This pairing provides broad-spectrum coverage against common Gram-positive pathogens as well as Gram-negative bacteria and synergic effect over *Staphylococcus* spp. (responsible of 2 of 3 PJI cases). Literature suggests that this combination offers optimal local release and mechanical stability, particularly when using commercial gentamicin-loaded cement supplemented with the manual addition of high doses of vancomycin per bone cement packet [3]. This strategy achieves local concentrations exceeding the minimum inhibitory concentration for typical pathogens while maintaining adequate mechanical resistance [2,4].

Recent research has explored further optimizing the pharmacokinetic profile of these spacers through multi-drug regimens. Multiple in vitro studies have demonstrated that adding a beta-lactam antibiotic to acrylic bone cement—such as cefazoline, imipenem–cilastatin or meropenem—significantly enhances the elution of vancomycin, with reported increases ranging from 30% to over 50% [6,7]. This synergistic effect is primarily attributed to increased porosity and alterations in the cement matrix when multiple antibiotics are incorporated [6]. While this phenomenon has been observed across various cement brands, it presents a clinical trade-off; higher antibiotic loads may compromise the mechanical integrity of the cement, though acceptable properties can be maintained by carefully adjusting dosages [7,8].

Despite these promising laboratory findings, a significant gap exists between in vitro data and clinical practice. Evidence regarding the clinical impact of beta -lactam/vancomycin combinations is limited to small-scale studies [9]. Furthermore, while systemic administration of certain beta-lactams with vancomycin has raised concerns regarding nephrotoxicity, the safety profile of these combinations in local delivery remains to be fully elucidated [10]. Consequently, there is currently no definitive evidence that the increased elution observed in the laboratory translates into improved patient outcomes.

Given the lack of large-scale comparative trials, further research is required to evaluate high-dose regimens in real-world settings to determine the optimal antibiotic combination and dosage for maximum bacterial eradication.

The primary objective of this study was to analyze and compare the clinical outcomes of patients treated with a multiple high-dose antibiotic-loaded cement (MHDALC) spacer against those receiving spacers prepared with alternative antibiotic combinations. This MHDALC spacer was prepared using a commercial bone cement pre-loaded with clindamycin and gentamicin, supplemented by the manual addition of ceftazidime and vancomycin. The secondary objective was to evaluate the efficacy and safety of a two-stage revision protocol for Gram-positive periprosthetic joint infections implemented at this institution.

## 2. Materials and Methods

### 2.1. Study Design and Participants

This retrospective, single-center, observational study was conducted at a tertiary hospital (Hospital General Universitario Gregorio Marañón). Patients included in the study were diagnosed with Gram-positive PJI following total knee or hip arthroplasty and underwent two-stage revision surgery between 2016 and 2023. Participants were identified from a prospectively maintained institutional database, and clinical data were retrieved through a retrospective review of electronic medical records.

The diagnosis of PJI was established according to the 2013 International Consensus Meeting criteria [11]. A standardized two-stage surgical protocol was applied in all cases, with all procedures performed by the senior surgeon. Patients were excluded if they did not undergo the specific two-stage protocol, failed to complete the second stage, or were lost to follow-up within the first postoperative year.

**Figure 1:**
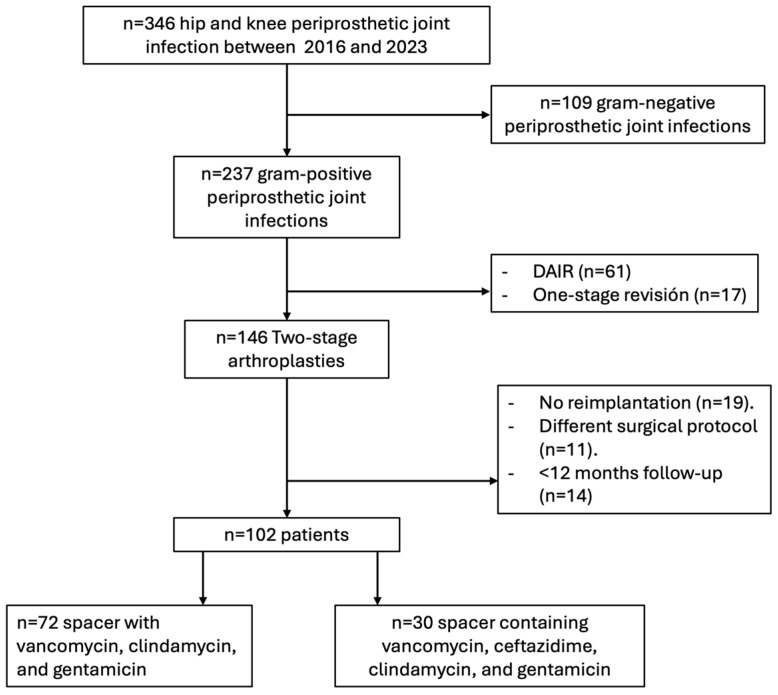
Patient selection algorithm and exclusion criteria. DAIR: debridement, antibiotics, and implant retention.

A total of 102 patients met the inclusion criteria. Within this cohort, 30 patients received a MHDALC spacer—prepared with commercial cement pre-loaded with clindamycin (1 g) and gentamicin (1 g), supplemented by the manual addition of ceftazidime (2 g) and vancomycin (4 g)—while the remaining 72 patients were treated with spacers incorporating alternative antibiotic combinations (Figure 1).

### 2.2. Ethical Statement

The study protocol was approved by the Scientific Ethics and Drug Research Committee of Gregorio Marañón General University Hospital. In accordance with Spanish legislation governing retrospective observational studies, all patients received written information regarding the study and were provided the option to decline participation. The requirement for written informed consent was waived by the ethics committee due to the study’s retrospective nature.

### 2.3. Treatment Protocol

Patients underwent a standardized two-stage revision procedure. During the first stage, all prosthetic components and intra-articular foreign materials were removed, followed by extensive mechanical debridement, synovectomy, and reaming of the endomedullary canal. The joint was then irrigated with 3 L of 0.9% physiological saline using low-pressure pulsatile lavage, followed by chemical debridement using 3% acetic acid, 5% hydrogen peroxide, and 10% povidone-iodine [12].

Microbiological samples were obtained following a strict institutional protocol: one synovial fluid sample via intra-articular aspiration and five tissue samples from standardized sites (synovial membrane, periarticular bone, and the bone–implant interface). All retrieved prosthetic components underwent sonication. After preparation, the joint was irrigated again with 3 L of saline, and all surgical instruments and drapes were replaced.

The spacer was prepared using commercial Copal G + C^®^ (Heraeus Medical GmbH, Wehrheim, Germany), which contains 1 g of gentamicin and 1 g of clindamycin per 40 g of cement. During the mixing phase at atmospheric pressure, 4 g of vancomycin and 2 g of ceftazidime were manually incorporated. Typically, three bags (120 g) of cement were used per case—two for the femur and one for the tibia—resulting in total doses of 12 g of vancomycin, 6 g of ceftazidime, 3 g of clindamycin, and 3 g of gentamicin. In the control group, the spacers were prepared in the same manner, but using only 4 g of vancomycin added to the Copal G + C cement.

**Figure 2:**
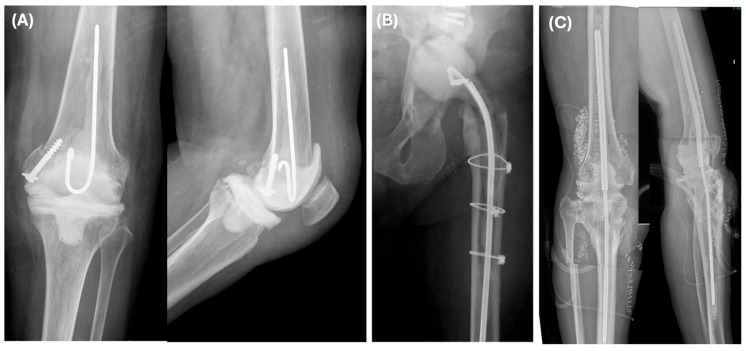
Radiographic examples following the first stage of revision surgery: articulated knee spacer (**A**), articulated hip spacer (**B**), and static knee spacer (**C**) used in a case of extensive bone loss.

The choice between an articulated or static spacer was based on bone stock and ligamentous integrity. Articulated knee spacers (Figure 2A) were either hand-molded or, in cases of substantial bone loss, fashioned using a ball-and-socket technique [13]. For hip revisions (Figure 2B), the femoral head spacer was molded using the bulb of a washing syringe over the cephalic screw of a nail or a Kuntscher nail [14]. The choice between articulated or static spacers was at the surgeon’s discretion, considering factors such as residual bone stock, ligamentous integrity and extensor mechanism status (Figure 2C).

Postoperatively, empirical antibiotic therapy was initiated and later adjusted based on culture results in consultation with infectious disease specialists. After a parenteral course of one week, patients transitioned to oral therapy. Reimplantation (second stage) was performed after an antibiotic-free interval of 2–3 weeks, provided clinical and laboratory markers (CRP and ESR) indicated infection eradication.

The second-stage procedure was performed under targeted prophylactic antibiotic coverage (daptomycin 10 mg/kg and ceftazidime 2 g IV) administered at least 30 min before skin incision. Following spacer removal, extensive debridement was undertaken, and at least five deep tissue samples were collected for culture, including the removed spacer, which was submitted for sonication following the same protocol as in the first stage. The clinical relevance of any positive culture results was reviewed by a multidisciplinary musculoskeletal infection team (orthopedic surgeons and microbiologists), considering both the number of positive samples and the identified microorganisms.

### 2.4. Outcome Assessment

The primary outcome was the eradication of PJI, defined as the absence of clinical or laboratory signs of infection and no meeting of treatment failure criteria (reoperation for sepsis, PJI-related death, or need for chronic suppressive therapy). Secondary outcomes included spacer exchange due to persistent infection, positive spacer cultures, and surgical or systemic complications. Joint function was assessed using the Harris Hip Score (HHS) for patients undergoing hip surgery and the Knee Society Score (KSS) for those undergoing knee surgery, with evaluations performed preoperatively, at one-year post-reimplantation, and at the final follow-up.

### 2.5. Statistical Analysis

Descriptive statistics were used to summarize baseline characteristics. Categorical variables were expressed as frequencies and percentages, while continuous variables were reported as means with standard deviation (SD) or medians with interquartile range (IQR). Group differences were analyzed using chi-squared or Fisher’s exact tests for categorical data. For continuous variables, the Kolmogorov–Smirnov test assessed normality; normally distributed data were compared using the Student’s *t*-test, and non-parametric data using the Mann–Whitney U-test. Treatment outcomes were estimated using Kaplan–Meier survival curves and compared via the log-rank test. A *p*-value < 0.05 was considered statistically significant. Analysis was performed using SPSS version 25.0 (IBM Corp., Chicago, IL, USA).

## 3. Results

### 3.1. Baseline Characteristics and Microbiology

A total of 104 patients were included, with 30 (28.8%) treated with a vancomycin plus ceftazidime-loaded spacer and 74 (71.2%) in the control group. Mean age was similar between groups (69.2 ± 11.6 vs. 68.0 ± 15.2 years). The prevalence of major comorbidities, including diabetes (6.5% vs. 18.7%), chronic kidney disease (6.5% vs. 12.0%), immunosuppression (6.5% vs. 9.3%), cardiovascular disease, and oral anticoagulant use, did not differ significantly between cohorts (Table 1).

**Figure 3:**
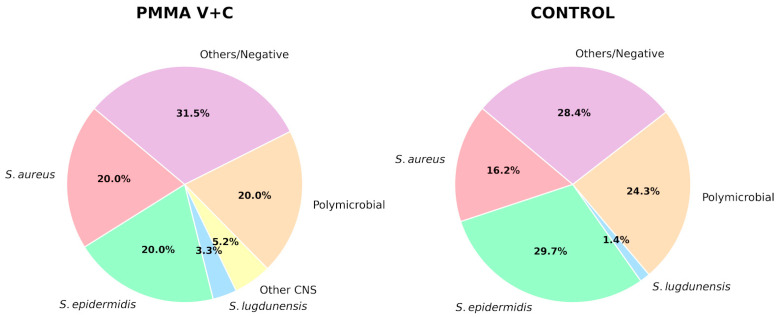
Distribution of causative microorganisms in the multiple high-dose antibiotic-loaded cement (PMMA V + C) group and the control group. *Staphylococcus* species, such as *S. aureus* and *S. epidermidis*, were the most frequently isolated pathogens in both cohorts.

The distribution of hip and knee arthroplasties was comparable, as was the proportion of previous aseptic revisions, which was numerically higher in the vancomycin plus ceftazidime group (51.6% vs. 34.7%; *p* = 0.406). Microbiological profiles were similar, with *Staphylococcus aureus* and coagulase-negative staphylococci being the most frequently isolated pathogens; polymicrobial infections accounted for approximately one quarter of cases (20% vs. 24.3%) (Figure 3). Infection type according to the Tsukayama classification showed no significant differences between groups (Table 1).

### 3.2. Clinical Outcomes

**Figure 4:**
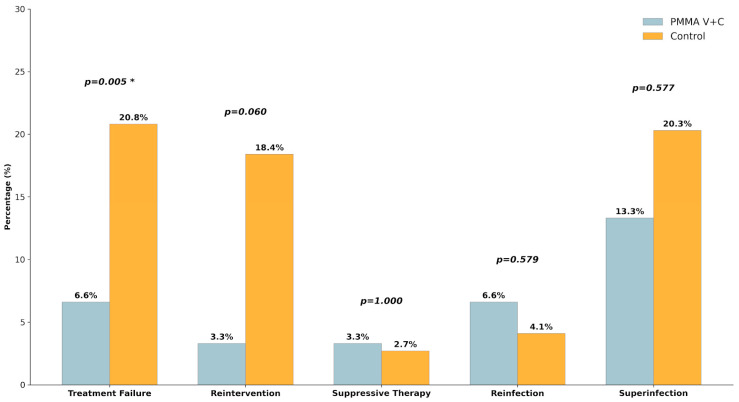
Comparison of treatment outcomes between the multiple high-dose antibiotic-loaded cement (PMMA V + C) group and the control group. The graph displays the percentages for treatment failure, reintervention, suppressive therapy, reinfection, and superinfection. The PMMA V + C group showed a significantly lower rate of treatment failure compared to the control group (6.6% vs. 20.8%; *p* = 0.005). Asterisks (*) indicate statistical significance.

Treatment outcomes differed according to the antibiotic-loaded spacer regimen. Patients treated with vancomycin plus ceftazidime had a significantly shorter time to reimplantation compared with controls (9.1 ± 5.6 vs. 17.8 ± 12.7 weeks; *p* = 0.001). Importantly, treatment failure was significantly lower in the vancomycin plus ceftazidime group (2/30, 6.6%) than in the control group (15/72, 20.8%; *p* = 0.005). Rates of positive cultures at second-stage reimplantation, infection persistence, and superinfection were comparable between groups, and follow-up duration did not differ significantly (Table 2, Figure 4).

Both study groups showed a functional improvement compared to baseline values, although no statistically significant differences were observed between the PMMA V + C and control cohorts at any of the follow-up intervals (Table 2).

**Figure 5:**
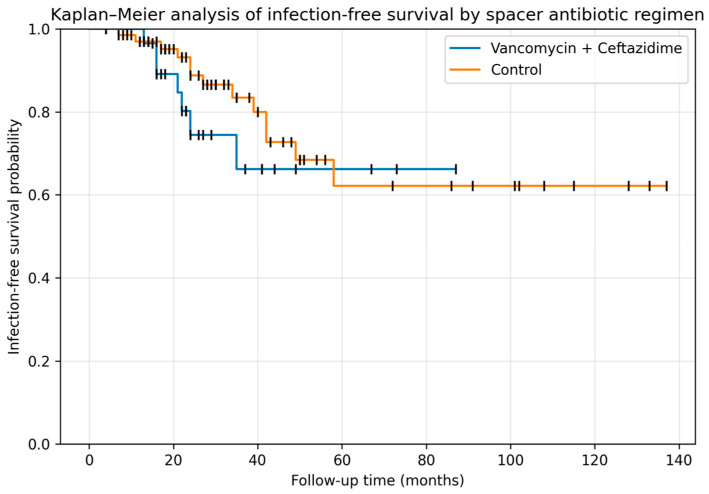
Kaplan–Meier curves showing infection-free survival according to antibiotic-loaded spacer regimen. Treatment failure was considered the event. Vertical tick marks indicate censored observations, including patients who died during follow-up.

Infection-free survival was analyzed using Kaplan–Meier estimates (Figure 5). Patients treated with vancomycin plus ceftazidime-loaded spacers showed a higher infection-free survival throughout follow-up compared with the control group; however, this difference did not reach statistical significance (log-rank test, *p* = 0.36).

### 3.3. Mechanical and Surgical Complications

**Figure 6:**
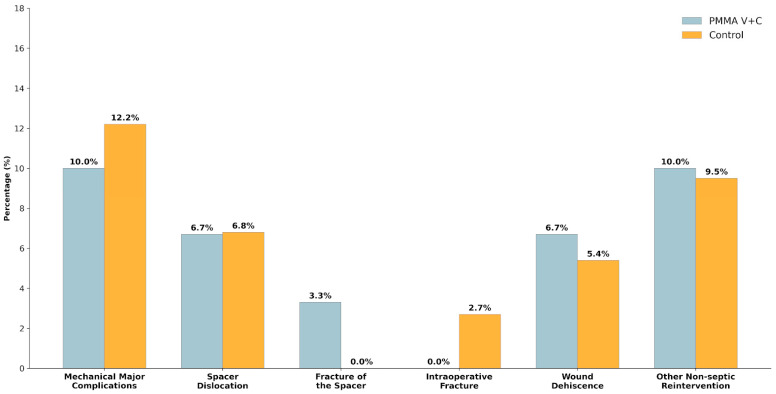
Comparison of surgical and mechanical complications between treatment groups. All differences observed regarding mechanical major complications, spacer dislocation, spacer fracture, intraoperative fracture, wound dehiscence, or other non-septic reinterventions were non-significant.

Overall, surgical complications were infrequent. Mechanical major complications were more commonly observed in hip spacers than in knee spacers, although this difference did not reach statistical significance (*p* = 0.552) (Table 3). Spacer dislocation was the most frequent, whereas spacer fracture and intraoperative fracture were rare events. The incidence of wound dehiscence and other non-septic reinterventions was low and comparable across groups. When complications were analyzed according to the antibiotic-loaded spacer regimen (vancomycin plus ceftazidime-loaded spacers vs. control), no significant differences were observed (Table 4, Figure 6).

## 4. Discussion

These findings reveal that an MHDALC spacer—incorporating clindamycin, gentamicin, vancomycin, and ceftazidime—enhanced clinical outcomes for Gram-positive PJI in this patient cohort. Primary findings reveal a three-fold reduction in the treatment failure rate in the MHDALC group (6.6%) compared to the control cohort (20.8%; *p* = 0.005). It is important to note that while the difference in infection-free survival probability between groups did not reach statistical significance in the Kaplan–Meier analysis (*p* = 0.36), the substantial reduction in the overall clinical failure rate suggests a superior antimicrobial efficacy of the MHDALC protocol. We hypothesize that failures in the MHDALC group tend to occur earlier and are primarily driven by new infections (superinfections), whereas failures in the control group often occur later and are characterized by the reactivation of the original pathogen (Figure 3). Therefore, while the survival analysis should be interpreted with caution, the overall trend across outcomes suggests that MHDALC spacers may contribute to improved infection control.

Beyond infection eradication, the MHDALC strategy was associated with a drastic reduction in the time to second-stage reimplantation, which decreased from an average of 17.8 weeks in the control group to just 9.1 weeks in the MHDALC group (*p* = 0.001). These clinical gains were achieved without compromising the structural integrity of the spacer; the incidence of mechanical failures remained low and comparable between both groups (*p* = 1.00), despite the higher antibiotic load. These findings suggest that the local antimicrobial environment provided by the MHDALC spacer not only facilitates more effective bacterial clearance but also allows for a significantly faster transition to definitive joint reconstruction.

The superior antimicrobial efficacy of the MHDALC group can be explained by two primary mechanisms: the increase in cement porosity and the optimization of antibiotic elution, particularly vancomycin [15]. The addition of multiple antibiotics in powder form, such as ceftazidime and vancomycin, significantly increases the porosity of the bone cement, which facilitates the diffusion and sustained release of the drugs [3]. Specifically, the incorporation of a small, highly hydrophilic molecule like ceftazidime dramatically enhances the porosity of the cement matrix; this, in turn, secondarily boosts the elution of larger molecules, such as vancomycin. This synergistic elution profile was demonstrated in vitro by Hsieh et al. [9], showing that the combination of vancomycin and ceftazidime in bone cement maintains effective antibacterial activity against common pathogens. Furthermore, recent clinical evidence has validated this approach; a comparative study by Hsieh et al. [16] found that the vancomycin-ceftazidime combination offers similar infection eradication rates to traditional regimens, such as vancomycin-tobramycin, but with significantly lower costs.

Literature supports that “home-made” manual mixing of vancomycin with gentamicin-premixed cement creates a more porous matrix than commercial formulations, allowing vancomycin—which is typically trapped within the acrylic matrix—to be released in significantly higher concentrations over a longer period [17]. A secondary effect is that, although ceftazidime is generally considered to lack clinical anti-staphylococcal efficacy, in vitro evidence suggests that the exceptionally high local concentrations achieved within the spacer environment may exhibit direct bactericidal activity against *Staphylococcus* species [18]. Also, polymicrobial infections and undetected Gram-negative pathogens are not uncommon in complex PJI cases, particularly in tertiary referral populations. Including a cephalosporin with Gram-negative coverage may therefore broaden the antimicrobial spectrum during the spacer phase.

Furthermore, the specific combination of Copal^®^ (clindamycin/gentamicin) with manually added vancomycin/ceftazidime provides a broader spectrum of coverage and addresses the challenge of biofilm formation [19]. Copal^®^ cement has demonstrated more extensive and prolonged antibiotic release and a higher capacity for biofilm inhibition compared to gentamicin-only formulations [20,21]. For vancomycin/ceftazidime combinations, in vivo data confirm joint fluid concentrations sufficient to inhibit both Gram-positive and Gram-negative pathogens [9]. By utilizing four synergistic antibiotics, the MHDALC spacer effectively reduces the risk of persistence and superinfection.

A critical benefit of the MHDALC protocol is the reduction of the interim period between stages from 27.8 to 9.1 weeks, which is vital for patient mobility. Complication rates, including mechanical failure and adverse events, are not typically increased with dual-antibiotic cements [22]. While high-dose vancomycin can reduce the compressive strength of cement, this study found no increase in mechanical complications such as fractures or dislocations in the MHDALC group. Functional outcomes, including pain and quality of life, typically do not differ significantly between single- and dual-antibiotic spacers, and this trend is expected to continue with additional antibiotics provided no mechanical complications occur [23].

The maximum recommended amount of antibiotic to maintain mechanical properties depends on the cement type. While literature suggests not exceeding 0.5 g of vancomycin per 40 g of cement to maintain standard ISO 5833 [24] compression resistance in prophylactic settings [25], loads of 1–2 g are common in temporary spacers where mechanical requirements are lower [26,27]. Other antibiotics, such as gentamicin, can be loaded up to 1.6 g without affecting shear strength significantly [28]. While general guidelines advise against exceeding 2 g of antibiotics per 40 g of cement to preserve mechanical integrity [29], this study substantially surpassed these thresholds. Moreover, manual mixing was conducted at atmospheric pressure rather than vacuum-mixing, potentially affecting the mechanical integrity of the cement [30]. Despite incorporating up to 8 g of antibiotics per 40 g batch, no significant increase in mechanical failure was observed. Several factors may explain this finding. First, antibiotic spacers are temporary devices designed to maintain joint space and deliver local antibiotics rather than to function as long-term load-bearing implants. Second, patients typically undergo protected weight-bearing and the duration of spacer implantation is limited until reimplantation. These may explain why the high antibiotic loading did not translate into clinically relevant mechanical complications.

No systemic complications were identified in this cohort. Systemic absorption of local antibiotics like vancomycin and gentamicin from cement spacers can cause nephrotoxicity or Acute Kidney Injury (AKI), especially when doses exceed 3–3.6 g per batch of cement [31,32]. The incidence of AKI following spacer insertion has been reported as high as 27% in some series. Factors such as advanced age, diabetes, and pre-existing renal insufficiency further increase this risk. The American Academy of Orthopaedic Surgeons (AAOS) cautions that indiscriminate use of high-dose antibiotic cement can lead to consequences such as antimicrobial resistance and increased costs [33]. However, the safety profile observed in this study suggests that the MHDALC regimen is clinically viable when managed by a multidisciplinary team.

This study has several limitations that must be addressed. Primarily, its retrospective design relies on the accuracy of electronic medical records and institutional databases, which inherently limits the level of evidence and carries a risk of data omission. An important limitation of this study is the presence of selection bias, as a substantial proportion of patients (40.4%) were complex tertiary referrals with previous failed surgical treatments. These cases are typically associated with more resistant organisms, compromised soft tissues, and multiple previous surgeries, all of which are recognized risk factors for treatment failure in PJI. Consequently, the inclusion of such high-risk cases may have diluted the observed treatment effect and potentially underestimated the benefits of the MHDALC protocol. In a population consisting predominantly of primary PJI cases, the magnitude of benefit could therefore be greater than that observed in this cohort.

Another significant constraint is the lack of exact cement and antibiotic quantification for each patient. Because spacers were manually customized to match the joint’s anatomy, they were not weighed in the operating room. We relied on the standardized concentration of antibiotics per cement pack, which does not account for individual variations in spacer volume or surface area. Additionally, the limited sample size of the MHDALC group (*n* = 30) reduces the statistical power of the study, likely explaining why clinically relevant trends—such as the differences in Kaplan–Meier survival curves—did not reach statistical significance.

Finally, there was a lack of standardization regarding the duration of interim antibiotic therapy and the timing of the second-stage surgery. These clinical decisions, as well as the transition from intravenous to oral therapy, were made at the discretion of a multidisciplinary team, introducing protocol heterogeneity and subjective variables that were not controlled for in this analysis.

## 5. Conclusions

In conclusion, the MHDALC spacer containing clindamycin, gentamicin, ceftazidime, and vancomycin is a safe strategy that may improve infection control in Gram-positive PJI. Although the survival analysis did not demonstrate statistical significance, this protocol was associated with a lower overall failure rate and a shorter time to reimplantation without compromising mechanical stability or increasing surgical complications. These findings suggest a potential clinical benefit, particularly in complex or high-risk cases. However, while these results are promising, further randomized clinical trials are required to confirm these findings and define the optimal antibiotic combinations and dosing strategies for this approach.

## Figures and Tables

**Table 1:** Baseline characteristics of patients stratified according to antibiotic-loaded spacer composition (control vs. vancomycin plus ceftazidime). Univariate analysis.

	All	PMMA V + C ^5^	Control	*p*-Value
N patients	102	30 (28.8%)	72 (71.2%)	
General characteristics				
Male Sex (%)	42 (40.4%)	13 (43.3%)	29 (39.2%)	0.696
Age in years (SD ^1^)	68.4 (±14.2)	68.2 (±11.6)	68.5 (±15.2)	0.418
Comorbidities				
Diabetes	16 (15.4%)	2 (6.7%)	14 (18.9%)	0.143
Chronic kidney disease	11 (10.6%)	1 (3.3%)	10 (13.5%)	0.171
Immunosuppressants	9 (8.7%)	2 (6.7%)	7 (9.5%)	1.000
Ischemic cardiopathy	9 (8.7%)	2 (6.7%)	7 (9.5%)	1.000
Cardiac Insufficiency	12 (11.5%)	4 (13.3%)	8 (10.8%)	0.740
Oral anticoagulants	18 (17.3%)	5 (16.7%)	13 (17.6%)	0.912
Prosthesis/Background				
TKA ^2^	58 (55.8%)	16 (53.3%)	42 (56.8%)	0.750
THA ^3^	42 (40.4%)	14 (46.7%)	28 (37.8%)	0.406
Previous aseptic revision	42 (40.4%)	14 (46.7%)	28 (37.8%)	0.406
Tumoral reconstruction	7 (6.7%)	0 (0.0%)	7 (9.5%)	0.189
Infection characteristics				
Causative Microorganism				
*S. aureus*	18 (17.3%)	6 (20.0%)	12 (16.2%)	0.644
Methicillin resistant	5 (4.8%)	3 (10.0%)	2 (2.7%)	0.143
Methicillin sensitive	13 (12.5%)	3 (10.0%)	10 (13.5%)	0.752
*S. epidermidis*	28 (26.9%)	6 (20.0%)	22 (29.7%)	0.311
Methicillin resistant	15 (14.4%)	5 (16.7%)	10 (13.5%)	0.760
Vancomycin resistant	1 (1.0%)	0 (0.0%)	1 (1.4%)	1.000
*S. lugdunensis*	2 (1.9%)	1 (3.3%)	1 (1.4%)	0.496
Other CNS ^4^	1	1 (5.2%)	0	
Polymicrobial	24 (23.1%)	6 (20.0%)	18 (24.3%)	0.635
Methicillin resistant	12 (11.5%)	3 (10.0%)	9 (12.2%)	1.000
Tsukayama classification				
I	2 (1.9%)	0 (0.0%)	2 (2.7%)	1.000
II	14 (13.5%)	1 (3.3%)	13 (17.6%)	0.062
III	9 (8.7%)	0 (0.0%)	9 (12.2%)	0.056
IV	34 (32.7%)	9 (30.0%)	25 (33.8%)	0.709

^1^ SD = standard deviation; ^2^ TKA = total knee arthroplasty; ^3^ THA = Total hip arthroplasty; ^4^ CNS = coagulase negative staphylococci; ^5^ PMMA = polymethylmethacrylate cement. V + C = Vancomycin and Ceftazidime.

**Table 2:** Follow-up and Treatment outcomes stratified by antibiotic-loaded spacer regimen. Univariate and bivariate analysis.

	All	PMMA V + C ^3^	Control ^4^	*p*-Value
N patients	102	30	72	
Duration of antibiotic treatment, weeks (SD ^1^)	9.9 (±11.9)	7.7 (±4.6)	10.8 (±13.7)	0.096
Surgical management				
Articulated spacer	86 (82.7%)	28 (93.3%)	58 (78.4%)	0.088
Time to reimplantation, weeks (SD)	15.2 (±11.8)	9.1 (±5.6)	17.8 (±12.7)	0.001
Second stage—positive cultures	18 (17.3%)	3 (10.0%)	15 (20.3%)	0.263
Surgical complication	11 (25%)	6 (31.5%)	8 (33%)	1.000
Mechanical complication	12 (11.5%)	3 (10.0%)	9 (12.2%)	1.000
Infection persistence	18 (17.3%)	4 (13.3%)	14 (18.9%)	0.579
Treatment failure ^2^	17 (16.7%)	2 (6.6%)	15 (20.8%)	0.005
Dead	0	0	0	
Reintervention	13 (12.5%)	1 (3.3%)	13 (18.4%)	
Suppressive therapy	2 (1.9%)	1 (3.3%)	2 (2.7%)	
Responsible microorganism				
Reinfection	5 (4.9%)	2 (6.6%)	3 (4.1%)	0.579
Superinfection	19 (18.3%)	4 (13.3%)	15 (20.3%)	0.577
Treatment failure excluding tumoral reconstructions	12 (12.4%)	2 (6.6%)	12 (17.9%)	0.253
Follow up time, months (SD)	35.7 (±28.9)	29.9 (±19.3)	38.0 (±31.8)	0.364
Clinical Outcomes				
Harris Hip Score (HHS)				
Preoperative		42.1 (±10.5)	41.5 (±11.2)	0.803
1 year post-op		68.4 (±12.1)	67.1 (±13.4)	0.645
Final follow-up		72.3 (±11.8)	70.8 (±12.6)	0.578
Knee Society Score (KSS)				
Preoperative		44.2 (±9.8)	43.8 (±10.4)	0.857
1 year post-op		66.7 (±11.5)	65.2 (±12.8)	0.579
Final follow-up		70.5 (±10.9)	69.1 (±11.7)	0.573

^1^ SD = standard deviation; ^2^ Treatment failure (Not cured) is defined by: Reintervention because of septic reasons (persistence of infection, reinfection, or superinfection); PJI-related dead; Suppressive antibiotic therapy; ^3^ PMMA V + C = Vancomycin + ceftazidime-loaded spacer. ^4^ Control = Vancomycin loaded spacer.

**Table 3:** Surgical complications stratified by spacer location (hip vs. knee) and knee spacer design.

	All Spacers	Hip	Knee		*p*-Value ^1^
			Static	Articulated	
N patients	102	42	9	48	
Mechanical major complication	12 (11.8%)	6 (14.3%)	2 (22.2%)	4 (8.3%)	0.552
Spacer dislocation	7 (6.9%)	3 (7.1%)	1 (11.1%)	3 (6.2%)	1.000
Fracture of the spacer	1 (1.0%)	1 (2.4%)	0	0	0.420
Intraoperative fracture	2 (2.0%)	0	1 (11.1%)	1 (2.1%)	0.508
Wound dehiscence	6 (5.9%)	2 (4.8%)	1 (11.1%)	3 (6.2%)	1.000
Other non-septic reintervention ^2^	10 (9.8%)	2 (4.8%)	0	7 (14.6%)	0.296

^1^ *p*-values were calculated using Fisher’s exact test for comparison between hip and knee spacers. ^2^ Other non-septic reintervention included vascular complications and reoperations due to poor soft-tissue evolution during the follow-up period.

**Table 4:** Surgical complications according to antibiotic-loaded spacer regimen (control vs. vancomycin plus ceftazidime).

	All Spacers	PMMA V + C ^3^	Control	*p*-Value ^4^
N patients	102	30	72	
Mechanical major complication ^1^	12 (11.5%)	3 (10.0%)	9 (12.2%)	1.000
Spacer dislocation	7 (6.7%)	2 (6.7%)	5 (6.8%)	1.000
Fracture of the spacer	1 (1.0%)	1 (3.3%)	0	0.288
Intraoperative fracture	2 (1.9%)	0	2 (2.7%)	1.000
Wound dehiscence	6 (5.8%)	2 (6.7%)	4 (5.4%)	1.000
Other non-septic reintervention ^2^	10 (9.6%)	3 (10.0%)	7 (9.5%)	1.000

^1^ Mechanical major complications included spacer dislocation, spacer fracture, prosthesis dislocation, and intraoperative fracture. ^2^ Other non-septic reinterventions included vascular complications and reoperations due to poor soft-tissue evolution. ^3^ PMMA V + C = Vancomycin + ceftazidime-loaded spacer. ^4^ *p*-values were calculated using Fisher’s exact test.

## Data Availability

The original contributions presented in this study are included in the article. Further inquiries can be directed to the corresponding author.

## Abbreviations

The following abbreviations are used in this manuscript:

PJI	Periprosthetic joint infection
MHDALC	Multiple high-dose antibiotic-loaded cement
PMMA	Polymethylmethacrylate cement
AKI	Acute Kidney Injury
AAOS	American Academy of Orthopaedic Surgeons

## References

[1] Kurtz S.M., Ong K.L., Schmier J., Mowat F., Saleh K., Dybvik E., Kärrholm J., Garellick G., Havelin L.I., Furnes O. (2007). Future clinical and economic impact of revision total hip and knee arthroplasty. J. Bone Jt. Surg. Am..

[2] Anagnostakos K., Meyer C. (2017). Antibiotic Elution From Hip and Knee Acrylic Bone Cement Spacers: A Systematic Review. BioMed Res. Int..

[3] Lunz A., Schonhoff M., Omlor G.W., Knappe K., Bangert Y., Lehner B., Renkawitz T., Jaeger S. (2023). Enhanced Antibiotic Release From Bone Cement Spacers Utilizing Dual Antibiotic Loading With Elevated Vancomycin Concentrations. Int. Orthop..

[4] Lunz A., Knappe K., Omlor G.W., Schonhoff M., Renkawitz T., Jaeger S. (2022). Mechanical Strength of Antibiotic-Loaded PMMA Spacers in Two-Stage Revision Surgery. BMC Musculoskelet. Disord..

[5] Mensah L.M., Love B.J. (2021). A Meta-Analysis of Bone Cement Mediated Antibiotic Release: Overkill, but a viable approach to eradicate osteomyelitis and other infections tied to open procedures. Mater. Sci. Eng. C.

[6] Cerretani D., Giorgi G., Fornara P., Bocchi L., Neri L., Ceffa R., Ghisellini F., Ritter M.A. (2002). The in Vitro Elution Characteristics of Vancomycin Combined With Imipenem-Cilastatin in Acrylic Bone-Cements. J. Arthroplast..

[7] Sanz-Ruiz P., Paz E., Abenojar J., del Real J.C., Vaquero J., Forriol F. (2014). Effects of Vancomycin, Cefazolin and Test Conditions on the Wear Behavior of Bone Cement. J. Arthroplast..

[8] Paz E., Sanz-Ruiz P., Abenojar J., Vaquero-Martín J., Forriol F., Del Real J.C. (2015). Evaluation of Elution and Mechanical Properties of High-Dose Antibiotic-Loaded Bone Cement. J. Arthroplast..

[9] Hsu Y.H., Hu C.C., Hsieh P.H., Shih H.-N., Ueng S.W., Chang Y. (2017). Vancomycin and Ceftazidime in Bone Cement as a Potentially Effective Treatment for Knee Periprosthetic Joint Infection. J. Bone Jt. Surg. Am..

[10] Alosaimy S., Rybak M.J., Sakoulas G. (2024). Understanding Vancomycin Nephrotoxicity Augmented by β-Lactams: A synthesis of endosymbiosis, proximal renal tubule mitochondrial metabolism, and β-lactam chemistry. Lancet Infect. Dis..

[11] Parvizi J., Gehrke T., Chen A.F. (2013). Proceedings of the International Consensus on Periprosthetic Joint Infection. Bone Jt. J..

[12] Márquez-Gómez M., Díaz-Navarro M., Visedo A., Hafian R., Matas J., Muñoz P., Vaquero J., Guembe M., Sanz-Ruíz P. (2023). An In Vitro Study to Assess the Best Strategy for the Chemical Debridement of PJI. Antibiotics.

[13] MacAvoy M.C., Ries M.D. (2005). The ball and socket articulating spacer for infected total knee arthroplasty. J. Arthroplast..

[14] Sanz-Ruiz P., Calvo-Haro J.A., Villanueva-Martinez M., Matas-Diez J.A., Vaquero-Martín J. (2018). Biarticular total femur spacer for massive femoral bone loss. Arthroplast. Today.

[15] Frew N.M., Cannon T., Nichol T., Smith T.J., Stockley I. (2017). Comparison of the Elution Properties of Commercially Available Gentamicin and Bone Cement Containing Vancomycin With ‘Home-Made’ Preparations. Bone Jt. J..

[16] Lewis D.C., Blackburn B.E., Archibeck J., Archibeck M.J., Anderson L.A., Gililland J.M., Certain L.K., Pelt C.E. (2024). Similar Efficacy and Lower Cost Associated With Ceftazidime Compared to Tobramycin Coupled With Vancomycin in Antibiotic Spacers in the Treatment of Periprosthetic Joint Infection. J. Arthroplast..

[17] Hsieh P.H., Tai C.L., Lee P.C., Chang Y.-H. (2009). Liquid Gentamicin and Vancomycin in Bone Cement: A Potentially More Cost-Effective Regimen. J. Arthroplast..

[18] Jones R.N., Barry A.L., Thornsberry C., Gerlach E.H., Fuchs P.C., Gavan T.L., Sommers H.M. (1981). Ceftazidime, a pseudomonas-active cephalosporin: In-vitro antimicrobial activity evaluation including recommendations for disc diffusion susceptibility tests. J. Antimicrob. Chemother..

[19] Cara A., Ballet M., Hemery C., Ferry T., Laurent F., Josse J. (2021). Antibiotics in Bone Cements Used for Prosthesis Fixation. Front. Med..

[20] Ensing G.T., van Horn J.R., van der Mei H.C., Busscher H.J., Neut D. (2008). Copal Bone Cement Is More Effective in Preventing Biofilm Formation Than Palacos R-G. Clin. Orthop. Relat. Res..

[21] Neut D., de Groot E.P., Kowalski R.S., van Horn J.R., van der Mei H.C., Busscher H.J. (2005). Gentamicin-Loaded Bone Cement With Clindamycin or Fusidic Acid Added: Biofilm formation and antibiotic release. J. Biomed. Mater. Res. Part A.

[22] Boelch S.P., Jordan M.C., Arnholdt J., Rudert M., Luedemann M., Steinert A.F. (2017). Loading With Vancomycin Does Not Decrease Gentamicin Elution in Gentamicin Premixed Bone Cement. J. Mater. Sci. Mater. Med..

[23] Corona P.S., Barro V., Mendez M., Cáceres E., Flores X. (2014). Industrially Prefabricated Cement Spacers: Do Vancomycin- And Gentamicin-Impregnated Spacers Offer Any Advantage?. Clin. Orthop. Relat. Res..

[24] (2002). Implants for surgery—Acrylic resin cements.

[25] Bishop A.R., Kim S., Squire M.W., Rose W.E., Ploeg H.-L. (2018). Vancomycin elution, activity and impact on mechanical properties when added to orthopedic bone cement. J. Mech. Behav. Biomed. Mater..

[26] Kim S., Bishop A.R., Squire M.W., Rose W.E., Ploeg H.-L. (2020). Mechanical, elution, and antibacterial properties of simplex bone cement loaded with vancomycin. J. Mech. Behav. Biomed. Mater..

[27] Pelletier M.H., Malisano L., Smitham P.J., Okamoto K., Walsh W.R. (2009). The compressive properties of bone cements containing large doses of antibiotics. J. Arthroplast..

[28] Kilicoglu O., Koyuncu L.O., Ozden V.E., Bozdag E., Sunbuloglu E., Yazicioglu O. (2008). Effect of antibiotic loading on the shear strength at the stem-cement interface. Int. Orthop..

[29] Humez M., Citak M., Luck S., Linke P., Gehrke T., Paul C., Kühn K. (2025). Enhancing PMMA Cements With Manually Added Antimicrobial Agents. APMIS.

[30] Meyer J., Piller G., Spiegel C.A., Hetzel S., Squire M. (2011). Vacuum-mixing significantly changes antibiotic elution characteristics of bone cements. J. Bone Jt. Surg. Am..

[31] Judd H., Benito J., Pannu T.S., Villa J.M., Higuera C.A., Corces A. (2023). Nephrotoxicity Related to Antibiotic-Loaded Spacers in a 2-Stage Revision for PJI. Orthopedics.

[32] Thomas T.L., Kothari P.D., Baker C.M., Tarabichi S., Clark S.C., Goh G.S. (2024). High Incidence of Acute Kidney Injury Following Antibiotic-Loaded Spacer Insertion for PJI. J. Arthroplast..

[33] American Academy of Orthopaedic Surgeons (2019). Diagnosis and Prevention of Periprosthetic Joint Infections: Evidence-Based Clinical Practice Guideline.

